# American Medical Association

**Published:** 1857-04

**Authors:** 


### AMERICAN MEDICAL ASSOCIATION.

As will be seen from the circular of the Secretary, this body will convene in the City of Nashville, on Tuesday, the 5th of May, 1857. We feel a deep interest in the Association, and would urge upon all organizations entitled to representation, to send on delegates, that we may have really, what it purports to be, a *National* Medical Convention, not sectional or local, and not to be influenced by cliques, or to be managed for the advancement of a few.

To the proceedings of such a body properly constituted and permanently established, we look for the elevation of the medical profession in many ways.

We heartily agree in the propriety of the proposition originating, we believe, with the Nashville Journal, that the President of the body should be selected, upon the ground of merit alone, without reference to locality.

